# Temporal trends in cardiovascular disease and diabetes prevalence in the United States: assessing compression versus expansion

**DOI:** 10.1038/s41598-026-48747-1

**Published:** 2026-04-16

**Authors:** Alberto Arletti, Lorenzo Schiavon, Mattia Stival, Michele Marzulli, Gaia Bertarelli, Stefano Campostrini

**Affiliations:** 1 Department of Economics, Ca’ Foscari University of Venice, Cannaregio 873, 30100 Venice, Italy; 2 Department of Statistical Sciences, University of Padova, Via Cesare Battisti 241, 35121 Padua, Italy; 3 Department of Mathematics, University of Padova, Via Trieste, 63, 35131 Padua, Italy

**Keywords:** Aging, BRFSS, Cohort analysis, Morbidity compression, Socioeconomic inequalities, Diseases, Endocrinology, Health care, Medical research, Risk factors

## Abstract

Recent trends from Western countries indicate a stagnation of life expectancy gains, raising questions about the mechanisms that sustain healthy aging and morbidity compression. In this study, we focus on trends in cardiovascular diseases and diabetes and their determinants in the United States, as relevant contributors to potential morbidity compression and, consequently, to increases in healthy life expectancy. Using data from the Behavioral Risk Factor Surveillance System from 1990 to 2023, we apply a pseudo-panel approach to analyze morbidity patterns across birth cohorts in the United States. The models account for covariates such as BMI, smoking, ethnicity, gender, income, and education, including relevant nonlinearities and interactions to capture heterogeneity in disease risk. Findings show that, at comparable ages, more recent cohorts exhibit higher disease risk, for diabetes, a pattern that is not consistent with morbidity compression. These results are largely explained by the growing presence of behavioral and socioeconomic risk factors, such as overweight and low income, which disproportionately affect younger cohorts. Our study contributes to the growing literature documenting worsening population health trajectories in countries with advanced medical systems and highlights the need for public health strategies that address both behavioral and structural determinants to promote healthy aging.

## Introduction

Longevity remains one of the most fundamental indicators of population health, reflecting the combined effects of medical progress, social development, and behavioral change^1^. One influential framework for interpreting trends in healthy aging is the compression of morbidity^2^. According to this concept, improvements in prevention and healthcare should delay the onset of chronic conditions, concentrating morbidity into a shorter period toward the end of life, as observed until a few years ago in several countries (see, e.g., the distinct but illustrative cases of Taiwan and Austria in^3,4^). However, recent evidence indicates that this ideal pattern is weakening. Life expectancy gains in high-income countries are slowing or stagnating, and the number of years lived in good health (also referred to as health-adjusted life expectancy, HALE,^1^) has begun to decline in some countries. The World Health Organization^1^ reports that in the United States, life expectancy has plateaued, whereas HALE has shortened over the past decade, suggesting that Americans are living longer but spending a growing share of their lives in poor health. Reductions in healthy years often precede a slowdown or even reversal in longevity improvements^5^, raising concern that the progress achieved during the twentieth century may not be sustainable.

Authors have pointed out the lack of social and health services for large segments of the population as a direct cause of this deterioration in healthy aging^6^. Social health services, defined as services provided in the community that integrate health care and social support to promote well-being and address social needs^7^, are a key difference that explains the health gap between the United States and other Western countries such as in Europe or Japan^8^. At the same time, a decrease in later-life healthy years is closely linked to the rising burden of chronic diseases, which are the focus of the present study. In this regard, population aging, environmental factors, and lifestyle changes, particularly in diet and physical activity, have all contributed to higher rates of non-communicable diseases (NCDs) such as diabetes and cardiovascular diseases (CVDs). Although cardiovascular mortality has declined substantially in recent decades, the rate of improvement has slowed since 2010, partly due to the growing prevalence of obesity, hypertension, and diabetes^9–11^. Meanwhile, diabetes has become one of the fastest-growing chronic conditions worldwide, driven by increasing obesity and sedentary behavior. Note that the BRFSS does not distinguish between diabetes types; therefore, all subsequent references to diabetes in this manuscript pertain to both type I and type II diabetes. Findings and conclusions are driven mainly by type II prevalence, which accounts for the vast majority of diagnosed cases (90–95%, according to^12–14^). In the United States, Behavioral Risk Factor Surveillance System (BRFSS) and National Health and Nutrition Examination Survey (NHANES) data show that the prevalence of diabetes has increased steadily since the 1990s across most demographic groups^15,16^, following similar worldwide trends^17^. Although incidence estimates from the U.S. Centers for Disease Control and Prevention (CDC) suggest a decline since around 2008 after adjusting for age^18^, this trend should be interpreted with caution. The apparent decrease may partly reflect changes in the composition of ethnic groups^19^ and the use of broad age categories in standardization, which can mask demographic aging within those classes and thus underestimate the true incidence in older segments of the population. In parallel, forecasting and population-based studies continue to indicate that the total number of individuals living with diabetes is rising, despite these reported signals of declining incidence^19,20^. Those who have diabetes tend to live longer with the condition due to improved treatment and management, thereby increasing the total number of prevalent cases^19^. Moreover, the onset of diabetes is occurring earlier in life, extending disease duration within each cohort and compounding overall prevalence^21^. This dynamic is consistent with increased disease prevalence at younger ages, raising questions about whether recent trends are consistent with morbidity compression.

**Fig. 1:**
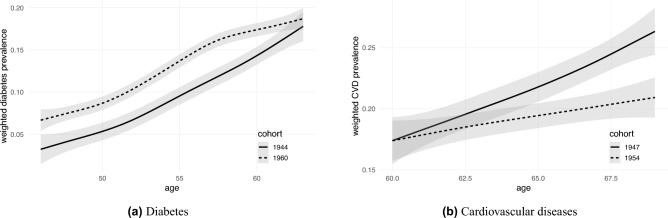
Smoothed weighted prevalence curves for diabetes and CVDs. Shaded areas represent smoothing standard deviation. For clarity, only two cohorts are shown. The remaining cohorts lie between these extremes, following the expected progression across birth cohorts.

These generational shifts in chronic disease dynamics are illustrated in Fig. 1, which displays the adjusted weighted prevalence of diabetes (left panel) and cardiovascular diseases (right panel) by age and birth cohort, as estimated from BRFSS data. The figures, which directly present prevalence computed using sample weights, differ in the x-axis to reflect the data composition in the sample as well as the most common age of onset and increased prevalence for both conditions. More recent cohorts exhibit higher prevalence levels and earlier onset of diabetes compared with earlier generations. While a reverse trend is observed in CVDs, it is remarkable how, in younger ages, the overlapping lines in the (b) plot indicate that there seems to be no improvement in disease prevalence for younger cohorts, possibly explaining the results in terms of healthy life.

A growing body of international evidence supports the hypothesis that the health of successive birth cohorts is worsening. Gimeno et al.^22^ define this trend as *Generational Health Drift*, observing that for several health outcomes, including obesity, mental ill-health, and diabetes, more recent cohorts born after 1946 exhibit higher prevalence of poor health when compared at the same age. Recent studies based on Italian data^23,24^ have investigated these patterns using survey analyses that relate morbidity to age, as well as to social and behavioral determinants, in order to disentangle their contribution to changes in population health over time. In the present work using US data, the focus is on social determinants of health, which refer to many broad factors that involve social and physical conditions of the environment in which people live and age, such as diet, poverty, education and more^25^.

In the United States, most research remains limited to aggregated age categories (e.g., 18–44, 45–64, 65+) and rarely investigates how social and behavioral determinants contribute to cohort-specific patterns of morbidity^18,19^. Although there are some signals of reduced disease incidence, the persistence of widening inequalities and risk factor expansion, particularly in obesity, suggests that successive cohorts may be entering midlife with higher baseline health risks. Understanding the determinants of chronic diseases is therefore crucial not only for explaining the observed rise in prevalence and the associated reduction in HALE, but also for informing effective prevention and policy responses.

In the Italian context, recent studies have examined individual morbidity in relation to age and social and behavioral determinants, aiming to disentangle their contribution to changes in population health over time^23,24^. In this study, we analyze the determinants of diabetes and cardiovascular diseases in the United States between 1990 and 2023 using data from the Behavioral Risk Factor Surveillance System [BRFSS,^26^] the largest program to provide state-representative information on health behaviors and condition through repeated cross sectional surveys. The BRFSS offers a unique opportunity for this analysis as it combines behavioral and chronic condition information at the individual level: an integration rarely available in socioeconomic or administrative datasets. Building on this richness, we apply a pseudo-panel framework inspired by previous morbidity studies using repeated cross-sectional data, such as Stival, Schiavon, and Campostrini^27^. By examining the role of birth cohort membership alongside socioeconomic and behavioral factors, such as income, education, BMI, smoking, and ethnicity, we examine patterns that may inform on morbidity compression or expansion. A positive cohort effect, indicating higher disease probability among more recent cohorts when controlling for other determinants, would reflect deteriorating generational health. Conversely, a null or negative cohort effect accompanied by increasing prevalence would suggest that population-level increases in disease prevalence are primarily driven by the broader diffusion of adverse risk factors, rather than by declines in medical or technological progress^28^. The term technological progress is used to refer to improvements in medical knowledge and health systems in a broad sense, including more effective pharmacological treatments, acute disease management, preventive screening, as well more effective health policies^29^. By diffusion of adverse risk factors, instead, we refer to changes in the population distribution across cohorts of all characteristics that increase disease probability, such as sedentary lifestyles, or worsening socioeconomic conditions^30^. By disentangling these mechanisms, this research contributes to a better understanding of the ongoing stagnation in U.S. healthy longevity and provides empirical evidence relevant for public health planning and preventive strategies.

## Methods

### Data and cohort design

We analyze the determinants of diabetes and cardiovascular disease (CVD) in the U.S. population, focusing on their evolution over time. Our data source is the BRFSS, an annual, nationally representative cross-sectional survey collecting information on health behaviors, chronic conditions, and preventive service use through approximately 400,000 telephone interviews per year^31^. Diabetes status is based on a single questionnaire item indicating self-reported diagnosis by a health professional, available consistently since 1990, and that does not discriminate between type I and type II diabetes. Cases of diabetes during pregnancy are excluded. Presence of CVDs is defined as the self-reported diagnosis by a health professional of coronary heart disease, myocardial infarction, or stroke, and has been consistently collected since 2007. Our objective is to assess the role of health determinants to understand the contribution of social and behavioral risk factors, as well as changes in health across generations.

To avoid conflating age and period effects, we adopt a pseudo-panel approach, using birth cohorts rather than survey years as the temporal unit of analysis. As noted by^27^, using survey year as the time axis can bias estimates because it mixes age effects with secular trends such as medical or technological advances.

To illustrate the approach, Figure 4 of the Supplementary Materials shows how, in a scenario of marginal morbidity expansion, the age-prevalence curves based on birth cohorts (red) are steeper than those based on survey years (blue). This reflects the fact that year-based prevalence trends are partially attenuated because older respondents belong to earlier birth cohorts with lower baseline morbidity, which offsets the underlying age effect. Cohort-based analysis better isolates generational patterns of morbidity.

To ensure robust estimates, we restrict the sample to age and cohort windows with sufficient observations. For diabetes, we use BRFSS waves from 1990 to 2023 and include respondents aged 45–64, born between 1945 and 1959. For CVD, we use BRFSS waves from 2007 to 2023 and include respondents aged 60–69, born between 1947 and 1954. The age windows align with evidence that type 2 diabetes and CVD typically emerge in mid to late adulthood^32,33^ and follow prior research on non-communicable diseases^23,27^. The cohort years are selected accordingly to maximize coverage without introducing empty age-cohort combinations in the pseudo-panel dataset. This lead to a sample of 1,876,686 respondents for diabetes, and 720,390 for CVDs.

### Model specification

For each condition, we model the binary outcome $y_n \in \{0,1\}$ indicating whether respondent *n* reports ($y_n=1$) or not ($y_n=0$) a diagnosis. We specify a logistic regression model

1 $$\begin{aligned} \Pr (y_n = 1 \mid \boldsymbol{x}_n) = \pi (\boldsymbol{x}_n) = \frac{\exp (\boldsymbol{x}_n^\top \boldsymbol{\beta })}{1 + \exp (\boldsymbol{x}_n^\top \boldsymbol{\beta })}, \end{aligned}$$

s where $\boldsymbol{x}_n$ includes sex at birth, age, birth cohort, income, ethnicity, BMI, education, smoking status, and geographical region, together with selected interactions and nonlinear terms. Similar covariates have been used in Italian studies^23,27^, with BMI and ethnicity added here to reflect U.S. heterogeneity and BRFSS data availability. Age, cohort, and BMI were standardized and included in the models as continuous variables; all other covariates are categorical, with category definitions reported in Table 1.

**Table 1:** Definition of categorical variables.

Variable	Description	Categories
Female	Sex at birth	True, false
Ethnicity		White, Black, Hispanic, other ethnicity
Income	Yearly household income adjusted for inflation	In poverty (less than $\$25,000$ ), Rich (more than $\$50,000$), Other income, no information on income
Education	Highest attained	Some college or professional degree, College or above, High school or less
Smoker	Smoked at least 100 cigarettes	True, False
Region		Midwest, north-east, west, south

Annual household income is harmonized across survey years by adjusting for inflation and collapsing reported brackets into a three-level categorical variable using $\$25{,}000$ and $\$50{,}000$ as cutoffs, such that the thresholds are expressed in constant dollars and are comparable across years. Respondents with missing income form a further separate category, consistent with prior work documenting substantial non-response^34^ and its association with self-employment^35^. Individuals with missing values for other covariates (less than 5% for BMI, less than 4% for ethnicity and less than 7.5% for all other combined) are excluded.

Because the number of possible interactions is large, we adopt a multistage model selection pipeline summarized in Figure 5 of the Supplementary Materials. We first split the data into a 75% training set and a 25% hold-out set for validation. Data splitting is employed to evaluate and compare alternative model specifications on observations not used for estimation, thereby limiting overfitting and providing an unbiased assessment of predictive performance, as routinely practiced in statistical modeling^36^. On the training set, we fit multivariate adaptive polynomial spline models for classification [PolyMars,^37^] relying on the polspline package in R^38^ to identify relevant nonlinearities and candidate interactions. PolyMars is a flexible nonparametric approach that iteratively explores a large space of model specifications, including nonlinear transformations (knots) and higher-order interactions. It evaluates alternative configurations through a data-driven procedure and selects the specification that optimizes model fit. This approach also highlights potential inflection points in cohort effects, which may correspond to shifts in morbidity trends. Relevant state effects are explored by fitting a further polynomial spline model including state as a categorical predictor.

In an initial stage, we aim to evaluate the inclusion of potential interactions among covariates. To do so, we use PolyMars applied to two different specifications of the predictor set, with and without state indicators, yielding candidate models that include data-driven nonlinearities and higher-order interactions. To further expand the scope of explored relationships and reduce the risk of omitting relevant effects, we complement this approach with a set of interactions motivated by prior literature, such as^39^, which are included in the pool of candidate terms for exploratory evaluation. Once a comprehensive set of candidate effects has been assembled, we proceed by employing two complementary data-driven selection methods to identify more parsimonious and empirically supported specifications. First, we apply a bi-directional stepwise regression and retain the ten best-performing models according to the Akaike Information Criterion (AIC). Second, we estimate Lasso models with cross-validation. Lasso is a method that performs both variable selection and regularization to improve prediction accuracy and model interpretability^40^. We select both the minimum-error (“min”) solution and the more regularized (“1se”) specification for Lasso. The resulting set of models—comprising PolyMars specifications, stepwise-selected models, Lasso solutions, and expert-defined models—is then compared in terms of selected variables and interactions. Effects that are consistently retained across the majority of models are included in the final specification. This procedure yields two nested models: a more comprehensive version (“expert large”) and a more parsimonious one (“expert mini”), which includes only effects selected by nearly all methods.

We compare the predictive performance of the expert models with stepwise regression, Lasso, PolyMars, random forests^41^, R package rforest], and gradient boosting^42^, XGBoost, R package xgboost], evaluating log-loss and AUC on the hold-out set. Results reported in Tables 1, 2 of the Supplementary Materials, indicate that the “expert mini” model achieves similar predictive accuracy compared to more complex models. We therefore adopt the “expert mini” specification for both diabetes and CVD for subsequent inference, offering a good balance between interpretability and predictive performance. The complete list of selected covariates, transformations, and interactions is reported in Tables 3, 4 of the Supplementary Materials, along with related estimated parameters and Wald confidence intervals.

Including interaction terms is essential to capture heterogeneous and non-additive relationships between covariates, which would be overlooked in models restricted to main effects only.

## Results

This section summarizes the estimated effects from the logistic models, with particular attention to generational trends in disease risk and to the influence of socioeconomic and behavioral factors when accounting for other variables in the model.

**Fig. 2:**
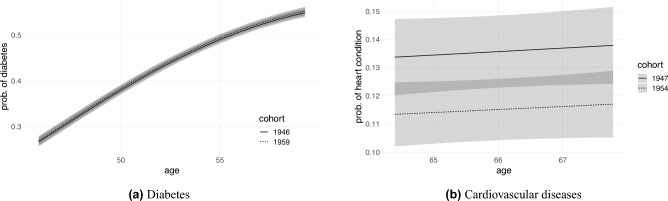
Estimated probability of diabetes and cardiovascular disease by age and cohort. Shaded areas represent 95% confidence intervals. Reference group is characterized as: male, smoker, living in the South, some college education, in poverty, black, BMI = 35. For clarity, only two cohorts are shown. The remaining cohorts lie between these extremes, following the expected progression across birth cohorts.

Logistic regression coefficients, 95% Wald confidence intervals, and *z*-tests at the 5% level are reported in Tables 3, 4 of the Supplementary Materials for both models. We are particularly interested in exploring how disease prevalence evolves across generations by assessing the cohort effect in explaining response variability. For diabetes, cohort coefficients are not statistically significant at $5\%$-level, and age-specific probabilities fully overlap across cohorts, as shown in the left panel of Fig. 2, confirming no measurable difference between the youngest and oldest cohorts when other covariates are held constant. For cardiovascular disease, younger cohorts show modestly lower prevalence at the same age, suggesting in this case slight morbidity compression, conditional on no variation in other covariates. The estimated effect indicates that each additional birth year corresponds to a relative risk reduction of $5\%$. Cohort effects resulted to be consistent across alternative model specifications.

**Fig. 3:**
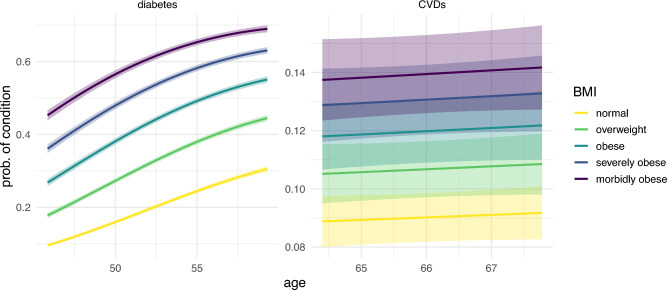
Diabetes and cardiovascular diseases morbidity curve for age and BMI levels. Shaded areas show 95% confidence intervals based on the logistic model. Reference group is characterized as: living in south, black, smoker, male, some college, in poverty, born in 1952. Note: y-axis differs between the two side-to-side figures.

This modest cohort effect may appear to contrast with the marked changes in disease prevalence observed in the U.S. population across cohorts when measured by interview year (see Fig. 1). This discrepancy may be explained by changes in other covariates over time, which strongly affect disease probabilities. Among these, as highlighted by Assaf et al.^28^, BMI plays a central role. BMI shows a strong nonlinear association with both conditions (Fig. 3). The linear term is strongly positive, while the quadratic term is negative, indicating a slight flattening of risk increases at very high BMI levels. Interactions with age and income are significant, but in all profiles, the probability of diagnosis rises sharply with BMI. For instance, at 48 years of age, considering the individual profile used in Fig. 3, moving from overweight (BMI $= 25$) to obese (BMI $= 30$) increases the predicted probability of diabetes by around 0.139 for diabetes at 59 years of age, and by 0.016 for cardiovascular disease at 65 years of age.

Given the ongoing obesity epidemic, these BMI-related effects may offset the modest cohort-based gains in cardiovascular morbidity reduction and contribute to the overall increase in diabetes prevalence. Therefore, it is worth also considering the cohort dynamics of covariates to assess their marginal impact over time.

**Fig. 4:**
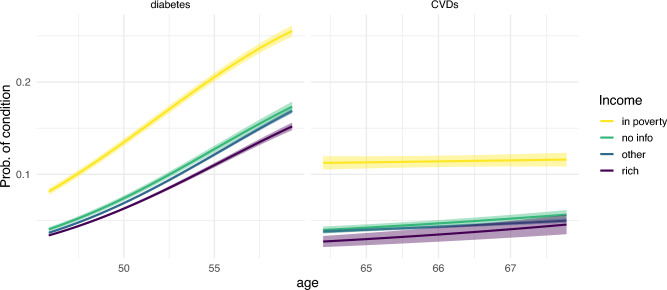
Estimated probability of diabetes and cardiovascular disease by age and household income. Shaded areas represent 95% confidence intervals. Reference group is characterized as: female, smoker, living in the South, born in 1952, white, some college education, BMI = 28.

**Fig. 5:**
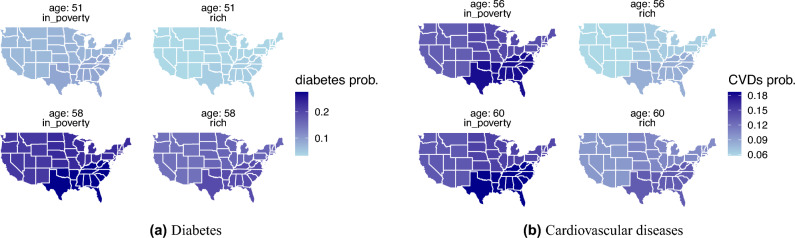
Estimated probability of diabetes and cardiovascular disease by age and the United States census region. Reference group is characterized as: male, smoker, some college education, white, BMI = 28, born in 1952.

During the model selection phase, income consistently emerged as one of the strongest predictors, interacting with most other covariates. Figure 4 displays the conditional effect of income across ages for both diseases for a reference subject. Respondents in the lowest income category ($\$25{,}000$ or less in inflation-adjusted yearly household income) show substantially higher probabilities of diabetes and cardiovascular disease compared to higher-income groups, holding other factors constant. For diabetes, the adverse effect of poverty becomes more pronounced with increasing age. Geographic disparities further amplify these social differences, with Southern states generally displaying higher morbidity levels, as seen in Fig. 5.

**Fig. 6:**
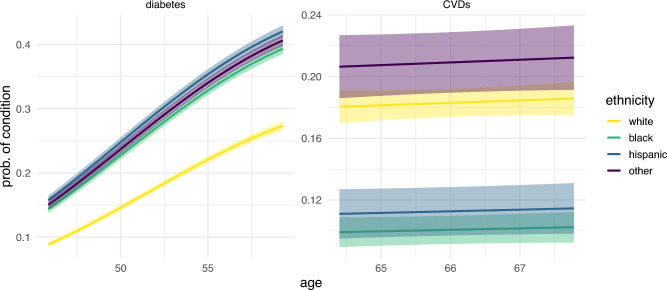
Estimated probability of diabetes and cardiovascular disease by age and ethnicity. Shaded areas represent 95% confidence intervals. Reference group is characterized as: male, smoker, living in the South, some college education, in poverty, born in 1952, BMI = 28. Note: y-axis differs between the two side-to-side figures.

Figure 6 shows predicted diabetes and cardiovascular disease probabilities by ethnicity. White respondents have a lower estimated probability of diabetes compared to Black and Hispanic respondents, with a relative reduction of $42\%$ ($\beta = -0.54$) when other covariates are held constant. Conversely, in the cardiovascular model, white ethnicity is associated with a higher risk compared to Black and Hispanic respondents, with a relative increase of $38\%$ ($\beta = 0.32$) under the same conditions.

Similar plots for the marginal probability of each condition by smoking behavior and education levels are presented in Figures 1, 2 in the Supplementary Materials.

## Discussion

In this study, we investigate how social and behavioral determinants influence two major non-communicable chronic diseases, namely diabetes and cardiovascular diseases (CVDs), within a selected segment of the aging U.S. population, to better understand healthy aging dynamics across cohorts. As outlined in the Introduction, social determinants of health refer to the social and environmental conditions in which individuals live and age, including factors such as diet, income, and education. To this end, we estimate two logistic regression models, one for each condition, using BRFSS data to evaluate the probability of diagnosis for individual respondents as a function of selected socio-demographic and behavioral covariates. The richness of the BRFSS dataset, which covers an extended time period and includes detailed geographic, socioeconomic, and behavioral information, strengthens the exploration of these determinants and long-term morbidity patterns across generations.

The results show no significant cohort effect for diabetes and only a modest one for cardiovascular diseases (CVDs). Since cohort effects capture differences in age-specific disease risk across generations, their limited magnitude indicates that younger cohorts do not experience substantially lower morbidity at comparable ages. As a result, there is little evidence of a marked postponement of disease onset across cohorts, and therefore only weak support for morbidity compression, primarily related to improvements in CVDs. This pattern is consistent with the hypothesis that technological and clinical advances have contributed to delaying CVD onset, as also reported in studies on the Italian population^24,27^. However, this modest compression effect, reflected in the lower CVD prevalence of more recent cohorts at older ages (Fig. 1b), seems to be only partly explained by technological and healthcare progress. Given that the estimated pure cohort effect is limited (Fig. 2b), much of the observed reduction in prevalence is likely associated with more favorable distributions of key protective factors across cohorts, such as lower smoking prevalence, higher levels of education, and changes in ethnic composition, as shown in Figures 3 and 4 of the Supplementary Materials. More broadly, these findings indicate that behavioral and socioeconomic factors are central to the evolution of morbidity across generations. Improvements in these factors may reinforce compression, as appears to occur for CVDs at older ages, whereas adverse trends may even offset medical advances and lead to an expansion of morbidity, as in the case of diabetes. This is consistent with the literature [see, e.g.,^43–45^], which identifies socioeconomic inequalities and lifestyle conditions as key determinants of variation in healthy aging across cohorts.

**Fig. 7:**
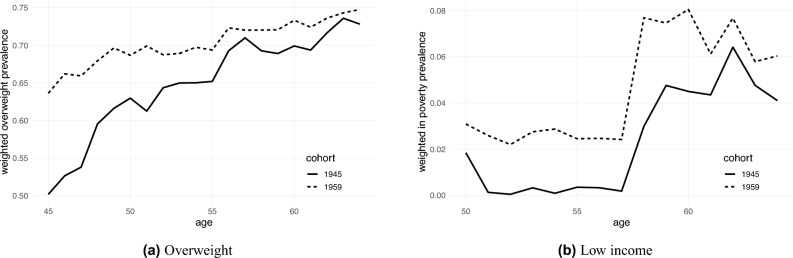
Weighted prevalence curves across cohorts for overweight (left) and low income (right; less than $\$25{,}000$ in inflation-adjusted yearly household income). For clarity, only two cohorts are shown. The remaining cohorts lie between these extremes, following the expected progression across birth cohorts. Weighting was carried out using the BRFSS-provided weights based on ethnicity, sex at birth, age and county.

Considering the strong influence that high BMI exerts on morbidity in both models, the sustained obesity epidemic^46,47^, illustrated by the increased overweight prevalence in the more recent cohort in panel (a) of Fig. 7, remains a major driver of diabetes prevalence and also a threat for CVDs. Obesity prevalence has increased steadily across younger cohorts, favoring earlier onset of disease and a cumulative rise in population burden.

In parallel, economic vulnerability has increased across cohorts, as shown by the higher share of respondents in low-income brackets in the panel (b) of Fig. 7. Results from our models (Fig. 4) indicate that individuals in the lowest income category face substantially higher probabilities of both diabetes and cardiovascular disease, consistent with extensive evidence linking socioeconomic disadvantage to metabolic and cardiovascular outcomes^48–50^. In the case of diabetes, the adverse effects of poverty also seem to intensify with age. Among older adults, particularly after retirement, limited financial resources further exacerbate vulnerability to chronic conditions through restricted access to healthy nutrition, opportunities for physical activity, and preventive care, all of which accelerate disease progression and undermine healthy aging. This pattern represents not only an epidemiological concern but also a broader social problem, as it reflects structural inequalities that persist or even widen in later life. The increasing diffusion of risk factors such as high BMI and low income contributes to higher disease prevalence at younger ages, which may in turn translate into an expansion rather than a compression of morbidity.

Diet quality is itself strongly linked to economic conditions in the United States: lower-income populations face greater barriers to accessing fresh and nutritious foods, particularly in areas with limited food availability or “food deserts”^51–53^. Obesity can also carry educational and occupational inequalities, serving as a proxy of a more complex socioeconomic background^54^. As other traditional behavioral risks, such as smoking and low education level, have declined across generations, as shown in Figure 3 of the Supplementary Materials, economic inequalities and diet quality seem to emerge as the main drivers of chronic disease prevalence and, consequently, of the reduction in HALE. While the present data show a sustained rise in obesity through the observation period, trends may shift post-2023. The use of glucagon-like peptide-1 (GLP-1) receptor agonists in the United States^55^ might alter future trajectories of obesity and related chronic conditions. Recent reports suggest the first decline in US obesity prevalence in decades^56^, a shift potentially linked to growing GLP-1 use. Although concerns remain regarding long-term sustainability^57^, widespread adoption of these therapies could reshape risk profiles in younger cohorts, with downstream effects on chronic disease in aging populations.

Regional disparities further compound the inequalities presented so far. As shown in Fig. 5, disease prevalence remains highest in the Southern states, even after controlling for individual socioeconomic characteristics. These regional effects likely reflect historical differences in welfare provision, preventive care programs, and public health expenditure^58,59^. For example, Duncan et al.^60^ have shown that location is responsible for changes in BMI and diet. States with weaker welfare infrastructures and lower investment in public health tend to exhibit higher burdens of chronic disease, consistent with evidence of a persistent social gradient in morbidity and mortality. Moreover, states with larger shares of the population living in remote or sparsely populated areas may experience reduced access to healthcare and diagnostic services, a concept supported by broader evidence on spatial disparities in health^61,62^. Demographic shifts, such as the national and international migration towards the Southern states of the country^63,64^, should be taken into account when comparing across cohorts between different geographical regions, as they might lead to different interpretation of the severity of the prevalence of conditions. Overall, our findings are consistent with previous research showing that individual health outcomes are deeply rooted in the institutional and policy environment in which people age, in accordance with the previous literature on the fundamental causes of health inequity^65–67^.

Ethnicity also plays a significant role in explaining health disparities, though its interpretation requires caution. Ethnicity is often correlated with socioeconomic and environmental factors^68^. Our models control for education, income, BMI, and region, allowing ethnicity to capture potential residual cultural or biological heterogeneity. Regarding the latter, it is interesting to note that our results seem consistent with patterns reported in the genetic literature, which describe lower genetic risk for cardiovascular disease but higher susceptibility to type 2 diabetes among African- and Hispanic-Americans compared with European-Americans^69–71^.

Overall, the results indicate a complex interplay between social, behavioral, and biological determinants shaping the health trajectories of U.S. cohorts. There is modest evidence of morbidity compression for cardiovascular diseases, while diabetes prevalence continues to rise across successive generations. Such results agree with similar findings in mortality and morbidity studies on diabetes and cardiovascular diseases^72,73^.

When interpreting the results, some limitations should be acknowledged. Firstly, it is important to note that our analysis did not cover all possible sources of inequality or increased morbidity in aging. Other influential factors, such as mental health^74,75^, social participation^76^, sexuality^77^, and gender identity^78,79^, also influence health outcomes and treatment disparities but were beyond the scope of this study. Second, although the BRFSS provides repeated cross-sectional data over a long time span, the analysis is constrained by the range of cohorts observed, limiting the ability to fully capture morbidity dynamics at older ages. Longer time series would allow for a more comprehensive assessment of late-life health trajectories and their policy implications. Third, the BRFSS is a cross-sectional survey and does not follow the same individuals over time. To mitigate this limitation, we adopt a pseudo-panel approach based on birth cohorts rather than survey years, which helps disentangle age and cohort effects. However, this approach relies on synthetic cohorts and cannot account for individual-level dynamics, such as within-person health transitions or selective survival. As a result, while cohort-based analysis improves the identification of generational patterns, it does not fully replicate the inferential strength of longitudinal data. Finally, the analysis does not include extrapolations to future years, which could illustrate how the observed trends might evolve over time. Despite these points, and although limited to two diseases, the analyses contribute to explain the life expectancy gap between the United States and many other countries. The persistence and expansion of socioeconomic and lifestyle inequalities suggest that structural conditions, rather than clinical shortcomings, are driving the stagnation and reduction of HALE in the United States. Policies aimed at reducing income disparities, improving dietary environments, and strengthening preventive health investment, particularly in disadvantaged regions, are therefore essential to reverse current trends and achieve genuine gains in healthy aging. As is well known, the United States is one of the countries that, both in absolute terms and as a percentage of GDP, spends the most on health services^80^. These results indicate that health outcomes are strongly shaped by social factors, suggesting that investments in health promotion and prevention may yield even greater benefits than those in clinical care. This view supports broader calls to strengthen the evidence base on the social determinants of health^81^ and aligns with sociological perspectives that frame health promotion as a collective process fostering equity and well-being^82^.

## Conclusions

This study adopted a cohort-based and multidimensional approach to examine morbidity dynamics in the United States, focusing on diabetes and cardiovascular diseases as key indicators of healthy aging. The added value of this analysis lies in considering together several interrelated factors, socioeconomic, behavioral, and generational, while explicitly incorporating a cohort perspective. This framework is crucial because it allows disentangling the contribution of demographic and social change from that of clinical and technological progress, providing a more comprehensive understanding of how morbidity evolves across generations.

Regression models show limited evidence of cohort-related morbidity reduction for cardiovascular diseases and no comparable improvement for diabetes. Although clinical and preventive advances have delayed disease onset and improved survival, their effects remain modest and are counteracted by adverse dynamics, potentially explaining the lack of substantial improvement in HALE. The results indicate that the generational dynamic of prevalence is largely driven by social and behavioral determinants, which in diabetes seems to offset the benefits of medical progress. At the same time, regression estimates further highlight that low income and high BMI consistently increase disease probability, showing that structural inequalities continue to shape health trajectories across U.S. cohorts. These findings support prior evidence that the lack of robust social health measures contributes to the stagnation of U.S. longevity relative to other high-income countries. The dynamic of morbidity can be assessed along multiple dimensions, our analysis focuses on prevalence patterns, which indicate rising burden at younger ages. In particular, for diabetes, these findings seem consistent with an expansion, rather than compression, of morbidity.

Despite focusing on only two chronic diseases, the study offers an important contribution to understanding the mechanisms underlying the U.S. lag in healthy aging. By combining a cohort-based framework with the analysis of individual determinants, it provides empirical evidence that can inform health policies aimed at reducing inequalities and promoting healthier lifestyles. The findings underscore that progress in medicine alone is insufficient to reverse the trend: effective health policy must also target the social and economic roots of disease.

Building on this contribution, future research could extend the analytical framework to additional chronic conditions to provide a broader perspective on morbidity dynamics. Another promising direction involves shifting from parameter estimation to prediction, using the model to estimate disease probabilities and forecast prevalence across subgroups defined by age, state, and cohort composition. Such extensions would help clarify whether changes in population structure translate into measurable improvements in health outcomes.

The evidence presented here is relevant beyond the United States. Similar dynamics of morbidity expansion have been documented in other advanced economies, suggesting that prevention-oriented strategies are universally applicable. The results confirm that investing in equality, improving healthy environments, and addressing metabolic risk factors such as obesity are fundamental for sustaining population health. Strengthening preventive and health-promoting measures alongside technological progress remains essential to achieve durable gains in HALE.

## Supplementary Information

Supplementary Information.

## Data Availability

The data used in this study are publicly available, de-identified data from the Centers for Disease Control and Prevention (CDC) Behavioral Risk Factor Surveillance System (BRFSS). These data can be accessed and downloaded from the official CDC BRFSS data portal: https://www.cdc.gov/brfss/annual_data/annual_data.htm.
