# Supplementary material for: Temporal trends in cardiovascular disease and diabetes prevalence in the United States: assessing compression versus expansion
Source: Sci Rep. 2026 Apr 16;16:17718. doi: 10.1038/s41598-026-48747-1 (PMC13247148; doi:10.1038/s41598-026-48747-1)
Supplement: Supplementary file 1 — Supplementary Information. [file 41598_2026_48747_MOESM1_ESM.pdf]

Temporal Trends in Cardiovascular Disease and Diabetes Prevalence in the United States: Assessing Compression versus Expansion

Alberto Arletti*[1], Lorenzo Schiavon[2,3], Mattia Stival[1], Michele Marzulli[1], Gaia Bertarelli[1], and Stefano Campostrini[1]

[1]Department of Statistics, Ca' Foscari University of Venice, Italy [2]Department of Statistical Sciences, University of Padova, Italy [3]Department of Mathematics, University of Padova, 35131, Italy

November 2025

Model	# Parameters	Log Loss	AUC
expert large	80	0.3239	0.7699
expert mini	38	0.3243	0.7692
lasso λ_{1se}	46	0.3243	0.7696
lasso λ_{min}	69	0.3239	0.7700
full linear	97	0.3238	0.7700
stepwise n=10	72	0.3240	0.7698
stepwise n=2	83	0.3238	0.7700
random forest	87	0.3380	0.7454
no interactions	17	0.3333	0.7635

Table 1: Summary of comparison of models by number of parameters, log loss, and AUC for diabetes.

Model	# Parameters	Log Loss	AUC
expert large	71	0.1831	0.7175
expert mini	57	0.1833	0.7165
lasso λ_{1se}	29	0.1843	0.7117
lasso λ_{min}	76	0.1831	0.7177
full linear	87	0.1831	0.7179
stepwise n=1	85	0.1831	0.7179
stepwise n=10	61	0.1831	0.7174
random forest	87	0.1882	0.6929
no interactions	17	0.1828	0.7143

Table 2: Summary of comparison of models by number of parameters, log loss, and AUC for CVs.

Term	Estimate	Std. Error	Pr(> $\|z\|$)	Sig.
(Intercept)	-1.0662	0.0215	0.0000	***
age_scl	0.3851	0.0111	0.0000	***
bmi_scl	0.8121	0.0066	0.0000	***
cohort_scl	0.0047	0.0027	0.0897	.
edu: high school or less	0.1650	0.0195	0.0000	***
edu: some college	0.1111	0.0215	0.0000	***
female	-0.0926	0.0125	0.0000	***
income: no info	-0.6998	0.0264	0.0000	***
income: other	-0.6110	0.0642	0.0000	***
income: rich	-0.6347	0.0206	0.0000	***
race: hispanic	0.1119	0.0117	0.0000	***
race: other	0.0536	0.0117	0.0000	***
race: white	-0.5452	0.0075	0.0000	***
region: Northeast	0.0032	0.0082	0.6989	
region: South	0.2148	0.0070	0.0000	***
region: West	-0.0949	0.0079	0.0000	***
smoker	0.1260	0.0048	0.0000	***
$I(\text{age\_scl}^2)$	-0.1088	0.0055	0.0000	***
$I(\text{bmi\_scl}^2)$	-0.0956	0.0007	0.0000	***
age_scl:bmi_scl	-0.0578	0.0034	0.0000	***
age_scl:income no info	0.0996	0.0153	0.0000	***
age_scl:income other	0.1269	0.0114	0.0000	***
age_scl:income rich	0.1107	0.0119	0.0000	***
bmi_scl:income no info	0.1219	0.0081	0.0000	***
bmi_scl:income other	0.0959	0.0061	0.0000	***
bmi_scl:income rich	0.2025	0.0064	0.0000	***
bmi_scl:region Northeast	0.0169	0.0066	0.0110	*
bmi_scl:region South	-0.0481	0.0056	0.0000	***
bmi_scl:region West	0.0205	0.0064	0.0014	**
edu: high school or less × income no info	0.3598	0.0272	0.0000	***
edu: some college × income no info	0.2123	0.0304	0.0000	***
edu: high school or less × income other	0.1465	0.0644	0.0229	*
edu: some college × income other	0.1224	0.0650	0.0594	.
edu: high school or less × income rich	0.0291	0.0939	0.7564	
edu: some college × income rich	0.1249	0.0245	0.0000	***
female × income no info	-0.0599	0.0197	0.0023	**
female × income other	-0.1065	0.0144	0.0000	***
female × income rich	-0.2011	0.0152	0.0000	***

Table 3: Logistic regression coefficients for the selected expert mini model for diabetes with standard errors, z values, p-values, and significance codes (p-value < 0.001 '***', < 0.01 '**', < 0.05 '*', < 0.1 '.'). Suffix "scl" indicates the scaled version of the continuous variable, with mean 0 and standard deviation equal 1.

The significance tests reported are unadjusted for multiple comparisons.

The coefficients of the selected model for diabetes are presented in Table 3. In the table, "scl" indicates the scaled version of the continuous variable, with mean 0 and standard deviation equal 1. Individuals with higher BMI show the strongest positive association with diabetes risk (0.8121, $p < 0.0001$), followed by older age (0.3851, $p < 0.0001$). Having only a high school degree or less (0.1650, $p < 0.0001$) or some college education (0.1111, $p < 0.0001$) is also linked with higher odds compared to higher educational attainment. Belonging to a Hispanic racial group (0.1119, $p < 0.0001$) or the "other" racial groups (0.0536, $p < 0.0001$) is associated with increased diabetes risk compared to the reference category. Living in Southern states (0.2148, $p < 0.0001$) also increases risk, as does being a smoker (0.1260, $p < 0.0001$). Some income-related interaction effects are strong positive predictors. For example, age interacting with no info on income (0.0996, $p < 0.0001$), age × other income (0.1269, $p < 0.0001$), and age × rich income (0.1107, $p < 0.0001$) all increase diabetes risk with age. Similarly, BMI × income no info (0.1219, $p < 0.0001$), BMI × income other (0.0959, $p < 0.0001$), and BMI × income rich (0.2025,

$p < 0.0001$) indicate that higher BMI amplifies risk across income groups. There are also education-income interactions, including high school or less × no income info (0.3598, $p < 0.0001$), some college × no income info (0.2123, $p < 0.0001$), high school or less × other income (0.1465, $p = 0.0229$), and some college × rich income (0.1249, $p < 0.0001$), which all increase diabetes risk. A smaller but significant positive effect is seen for BMI × Northeast states (0.0169, $p = 0.0110$) and BMI × West states (0.0205, $p = 0.0014$). On the other hand, several variables show negative or protective associations. Being female is associated with a lower risk (–0.0926, $p < 0.0001$). Individuals reporting no income information (–0.6998, $p < 0.0001$), other income categories (–0.6110, $p < 0.0001$), or rich income (–0.6347, $p < 0.0001$) also have significantly lower baseline risk compared to the reference income group (income < 25.000$). Belonging to the white racial group reduces risk (–0.5452, $p < 0.0001$), and living in the Western states similarly shows a protective effect (–0.0949, $p < 0.0001$). There are also significant negative quadratic terms, indicating non-linear relationships: age^2 (–0.1088, $p < 0.0001$) and BMI^2 (–0.0956, $p < 0.0001$), suggesting that risk increases at lower to moderate levels but plateaus or declines at extreme values. The age × BMI interaction is negative (–0.0578, $p < 0.0001$), meaning the combined effect of older age and higher BMI is somewhat attenuated compared to their individual effects. Additionally, BMI × living in Southern states shows a negative interaction (–0.0481, $p < 0.0001$). Finally, several interactions involving sex and income categories are protective: female × no income info (–0.0599, $p = 0.0023$), female × other income (–0.1065, $p < 0.0001$), and female × rich income (–0.2011, $p < 0.0001$), indicating that women in these income groups have lower diabetes risk relative to men. A few variables show weak or non-significant effects. Cohort (scaled) is marginally positive (0.0047, $p = 0.0897$), while region Northeast has no significant effect (0.0032, $p = 0.699$). Education × rich income for those with high school or less is also non-significant (0.0291, $p = 0.756$).

Term	Estimate	Std. Error	Pr(> $\|z\|$)	Sig.
(Intercept)	-2.9352	0.0733	0.0000	***
age_scl	0.0298	0.0333	0.3713	
bmi_scl	0.2273	0.0121	0.0000	***
cohort_scl	-0.1197	0.0244	0.0000	***
edu: high school or less	0.1781	0.0395	0.0000	***
edu: some college	0.1378	0.0429	0.0013	**
female	-0.0584	0.0436	0.1798	
income: no info	-0.7246	0.0918	0.0000	***
income: other	-0.8060	0.1367	0.0000	***
income: rich	-0.7797	0.0812	0.0000	***
race: hispanic	0.0209	0.1208	0.8626	
race: other	0.5398	0.0850	0.0000	***
race: white	0.3240	0.0613	0.0000	***
region: Northeast	-0.1103	0.0727	0.1293	
region: South	-0.1092	0.0503	0.0299	*
region: West	0.0363	0.0995	0.7154	
smoker	0.6990	0.0122	0.0000	***
I(bmi_scl^2)	-0.0317	0.0028	0.0000	***
age_scl:income no info	0.2789	0.0557	0.0000	***
age_scl:income other	0.2077	0.0398	0.0000	***
age_scl:income rich	0.4191	0.0463	0.0000	***
bmi_scl:income no info	0.1038	0.0197	0.0000	***
bmi_scl:income other	0.0621	0.0138	0.0000	***
bmi_scl:income rich	0.1663	0.0170	0.0000	***
cohort_scl:income no info	0.1172	0.0400	0.0034	**
cohort_scl:income other	-0.0347	0.0291	0.2329	
cohort_scl:income rich	0.0036	0.0340	0.9145	
edu: high school or less × income no info	0.4405	0.0581	0.0000	***
edu: some college × income no info	0.2796	0.0633	0.0000	***
edu: high school or less × income other	0.3819	0.1264	0.0025	**
edu: some college × income other	0.3116	0.1274	0.0145	*
edu: some college × income rich	0.2633	0.1215	0.0302	*
female × income no info	-0.2742	0.0419	0.0000	***
female × income other	-0.3851	0.0307	0.0000	***

Term	Estimate	Std. Error	Pr(> $|z|$)	Sig.
female × income rich	-0.6127	0.0375	0.0000	***
income no info × race hispanic	-0.0933	0.1126	0.4070	
income other × race hispanic	-0.1525	0.0815	0.0613	.
income rich × race hispanic	-0.1075	0.1194	0.3679	
income no info × race other	-0.1832	0.0968	0.0584	.
income other × race other	-0.1258	0.0691	0.0688	.
income rich × race other	-0.0691	0.0890	0.4372	
income no info × race white	-0.4089	0.0665	0.0000	***
income other × race white	-0.2913	0.0483	0.0000	***
income rich × race white	-0.3940	0.0657	0.0000	***
female × race hispanic	-0.2316	0.0713	0.0012	**
female × race other	-0.1559	0.0583	0.0075	**
female × race white	-0.4952	0.0408	0.0000	***
race hispanic × region Northeast	0.2042	0.1350	0.1304	
race other × region Northeast	-0.0233	0.1065	0.8269	
race white × region Northeast	0.0863	0.0750	0.2501	
race hispanic × region South	0.1053	0.1184	0.3739	
race other × region South	0.3200	0.0780	0.0000	***
race white × region South	0.3694	0.0527	0.0000	***
race hispanic × region West	-0.1175	0.1422	0.4088	
race other × region West	-0.3950	0.1157	0.0006	***
race white × region West	-0.1519	0.1012	0.1334	

Table 4: Logistic regression coefficients for the selected expert mini model for CVDs with standard errors, z values, p-values, and significance codes (p-value < 0.001 '***', < 0.01 '**', < 0.05 '*', < 0.1 '.').

The significance tests reported are unadjusted for multiple comparisons.

The coefficients of the selected model for cardiovascular diseases (CVDs) are reported in Table 4. Similar to the diabetes model, all continuous predictors (with suffix "_scl") are standardized to mean 0 and standard deviation 1. BMI shows a strong positive association with CVD risk (0.2273, $p < 0.0001$), confirming its relevance as a key metabolic determinant. Smoking is also one of the strongest predictors (0.6990, $p < 0.0001$). Education and income remain significant, with lower educational attainment, high school or less (0.1781, $p < 0.0001$) and some college (0.1378, $p = 0.0013$), associated with higher risk compared to college graduates. Individuals with incomplete or missing income information (–0.7246, $p < 0.0001$), other income categories (–0.8060, $p < 0.0001$), and rich income (–0.7797, $p < 0.0001$) exhibit lower baseline risk relative to the lowest income group. Cohort effects are negative and significant (–0.1197, $p < 0.0001$), suggesting a modest morbidity compression for younger generations once other covariates are controlled.

Interaction terms reveal several important patterns. Age interacts positively with income, with stronger effects for higher-income respondents, indicating that socioeconomic advantage amplifies age-related CVD risk. BMI interacts positively with income categories as well, reinforcing that the impact of adiposity on CVD risk is more pronounced in wealthier groups. Educational level and income combinations, such as high school or less × no income info (0.4405, $p < 0.0001$) and some college × no income info (0.2796, $p < 0.0001$), also increase risk. Sex and income interactions show consistently negative signs, implying that women in all income groups have lower CVD risk than men (female × income rich = –0.6127, $p < 0.0001$). Ethnic and regional interactions reveal additional heterogeneity: being of "other" race in Southern states (0.3200, $p < 0.0001$) or being White in the South (0.3694, $p < 0.0001$) significantly increases risk, while residing in Western regions mitigates it (race other × West = –0.3950, $p = 0.0006$). The quadratic term for BMI (–0.0317, $p < 0.0001$) indicates a nonlinear pattern where risk rises with BMI but plateaus at higher levels. Overall, the CVD model confirms that both behavioral (smoking, BMI) and socioeconomic factors (education, income, region) remain central to explaining cardiovascular morbidity across U.S. cohorts.

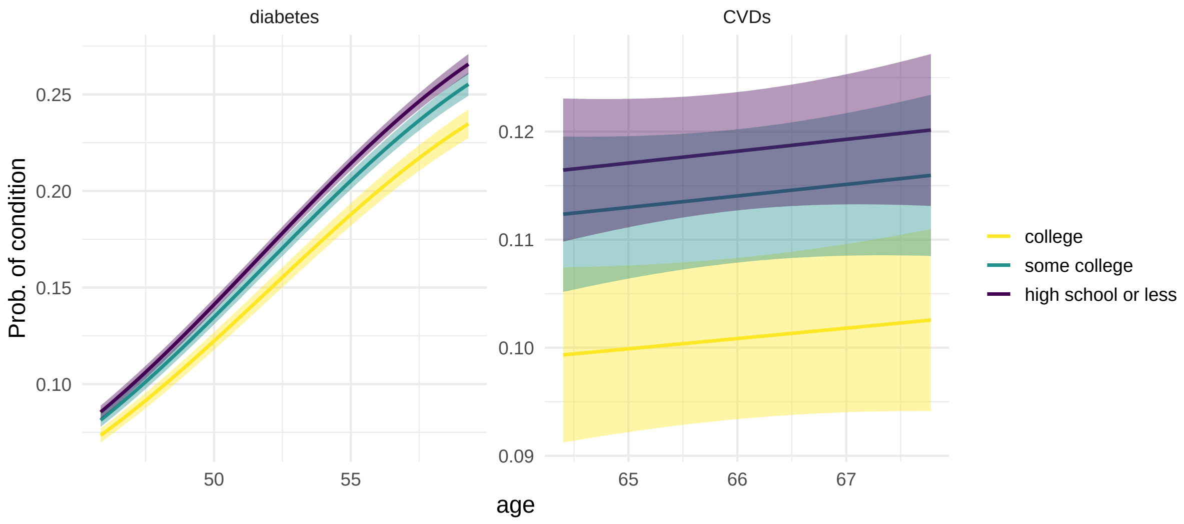

Figure 1: Estimated probability of diabetes and cardiovascular disease by age and education. Shaded areas show 95% confidence intervals based on the logistic model. Reference individual: living in south, in poverty, female, born in 1952, white, education: some college, BMI of 28.

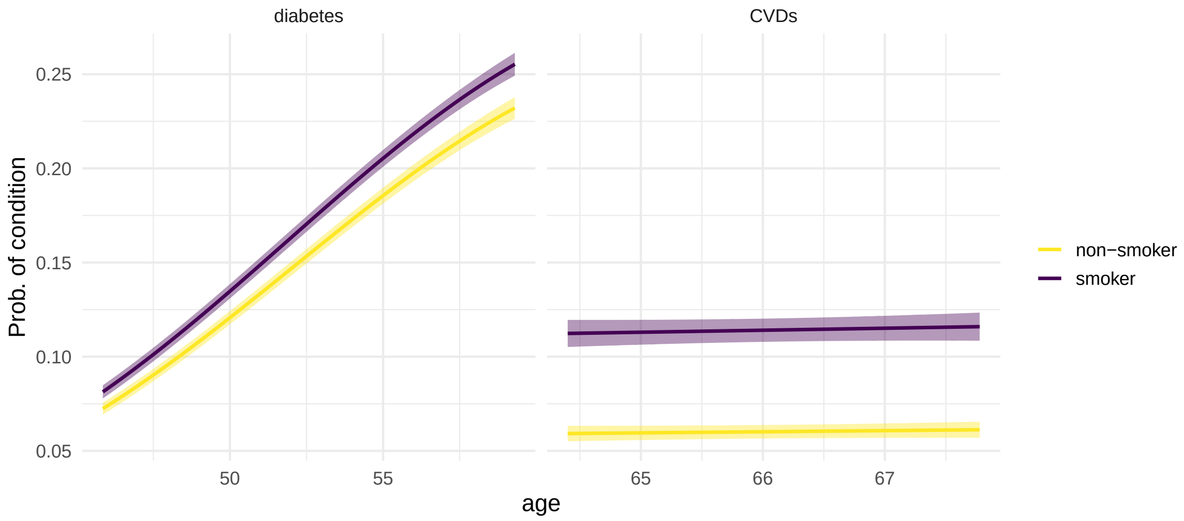

Figure 2: Estimated probability of diabetes and cardiovascular disease by age and smoking. Shaded areas show 95% confidence intervals based on the logistic model. Reference individual: living in south, smoker, female, born in 1952, white, in poverty, BMI of 28.

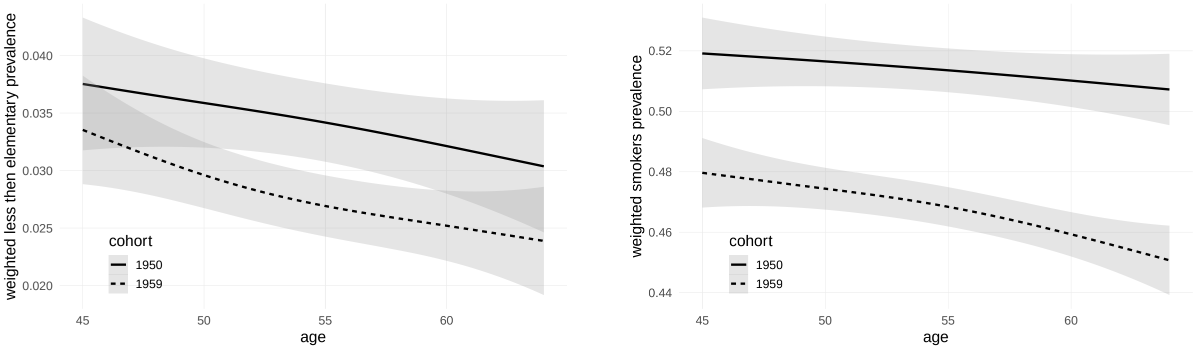

(a) Low Education (b) Smokers

Figure 3: Smoothed weighted prevalence curves for low education (attained less than elementary) and smokers (smoking or ever smoked 100 cigarettes). Shaded areas represent smoothing standard deviation.

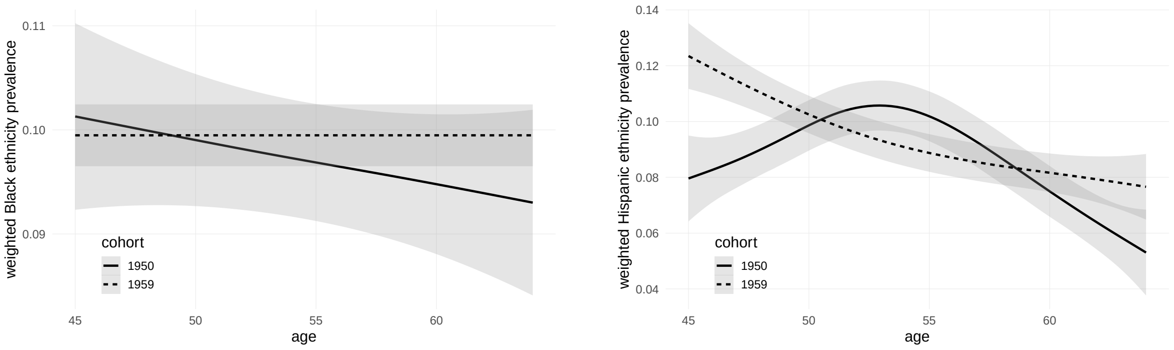

(a) Black Ethnicity (b) Hispanic Ethnicity

Figure 4: Smoothed weighted prevalence curves for Black and Hispanic ethnicity. Shaded areas represent smoothing standard deviation.

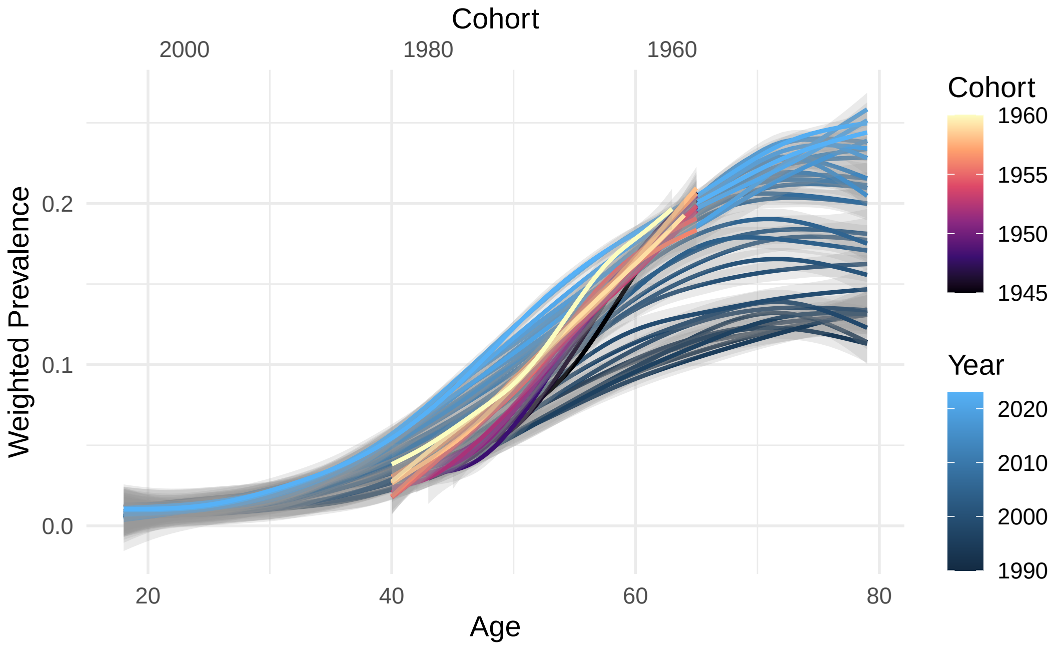

Figure 5: Diabetes weighted prevalence (y-axis) by age (x-axis), comparing cohort-based (red) and survey year-based (blue) estimates. The cohort-based (pseudo-panel) curves are steeper, as year-based prevalence estimates for older ages are attenuated by the inclusion of individuals from earlier, lower-risk cohorts. This illustrates how organizing the data by cohort helps reduce the confounding of age and period effects.

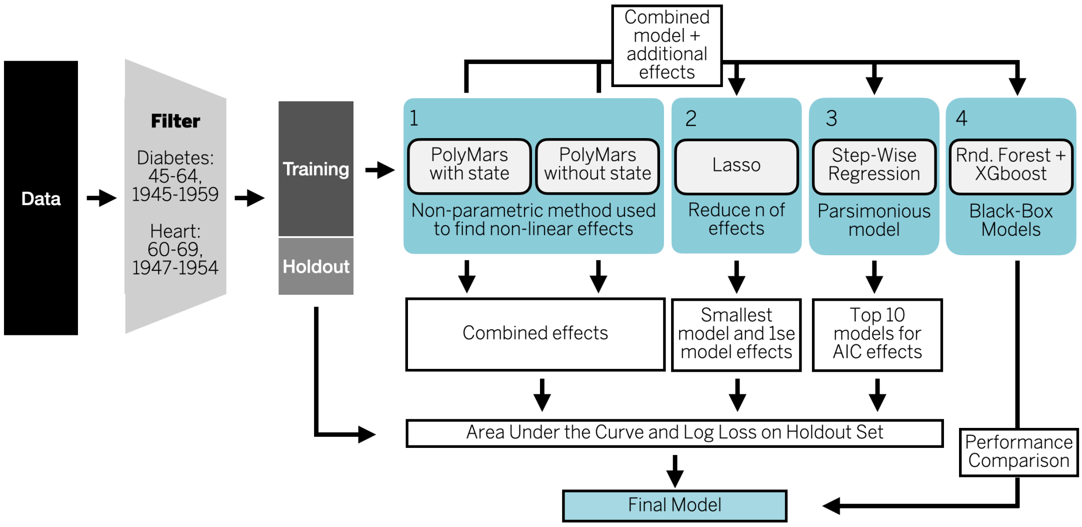

Figure 6: Schematic representation of the model selection pipeline
